# Establishment and characterization of patient-derived tumor xenograft using gastroscopic biopsies in gastric cancer

**DOI:** 10.1038/srep08542

**Published:** 2015-02-25

**Authors:** Yan Zhu, Tiantian Tian, Zhongwu Li, Zhiyu Tang, Lai Wang, Jian Wu, Yilin Li, Bin Dong, Yanyan Li, Na Li, Jianling Zou, Jing Gao, Lin Shen

**Affiliations:** 1 Department of Gastrointestinal Oncology, Key laboratory of Carcinogenesis and Translational Research (Ministry of Education), Peking University Cancer Hospital and Institute, Beijing, China; 2 Department of Pathology, Key laboratory of Carcinogenesis and Translational Research (Ministry of Education), Peking University Cancer Hospital and Institute, Beijing, China; 3 BeiGene (Beijing) Co., Ltd, China; 4 MyGenostics Inc. Beijing, China

## Abstract

The patient-derived tumor xenograft (PDTX) model has become the most realistic model for preclinical studies. PDTX models of gastric cancer using surgical tissues are reported occasionally; however, the PDTX models using gastroscopic biopsies, which are best for evaluating new drugs, are unreported. In our study, a total of 185 fresh gastroscopic biopsies of gastric cancer were subcutaneously transplanted into NOD/SCID (Nonobese Diabetic/Severe Combined Immunodeficiency) mice. Sixty-three PDTX models were successfully established (34.1%, 63/185) and passaged to maintain tumors *in vivo*, and the mean latency period of xenografts was 65.86 ± 32.84 days (11–160 days). Biopsies of prior chemotherapy had a higher transplantation rate (52.1%, 37/71) than biopsies after chemotherapy (21.9%, 25/114; *P* = 0.000). No differences were found between the latency period of xenografts and characteristics of patients. The pathological and molecular features of PDTX as well as chemosensitivity were highly consistent with those of primary tumors of patients. The genetic characteristics were stable during passaging of PDTX models. In summary PDTX models using gastroscopic biopsies in gastric cancer were demonstrated for the first time, and the biological characteristics of the PDTX models were highly consistent with patients, which provided the best preclinical study platform for gastric cancer.

The percentage of advanced gastric cancer (AGC) in China is very high with poor prognosis and high mortality^1^. The comprehensive treatment based on fluorouracil containing chemotherapy is the main strategy for AGC. Although the addition of targeted drugs (trastuzumab, apatinib, and ramucirumab)^2^,^3^,^4^ has improved the prognosis to some extent in recent years, the clinical outcome of AGC is not satisfactory due to the fewer therapeutic drugs and frequent drug resistance resulting from high heterogeneity and other mechanisms. As a consequence, developing new drugs and exploring the mechanisms of drug resistance are very urgent for AGC.

Currently, the most commonly used models for developing new drugs are *in vitro* cell lines or *in vivo* animal models established by injection of cell lines^5^,^6^. With the rapid progression of scientific research, the above models are unable to meet the clinical needs. It is well known that almost all cell lines are subcultured many times *in vitro* and lose most features of patients^7^,^8^. Moreover, the microenvironment of *in vitro* cell lines is completely different from primary tumors of patients due to lack of tumor-associated stroma and blood supply, and so on^9^. A kind of ideal model is needed for the preclinical study.

Patient-derived tumor xenograft (PDTX) models have become popular in the last several years with more advantages than cell line-based models^10^,^11^,^12^. Nowadays, most PDTX models are established by subcutaneously transplanting tumor tissues of patients into NOD/SCID (Nonobese Diabetic/Severe Combined Immunodeficiency) mice, and the biological characteristics of PDTX models are consistent with primary tumors of patients^13^,^14^,^15^,^16^. PDTX models from various tumors have been established, such as colorectal cancer^17^,^18^, breast cancer^15^,^19^, non-small cell lung carcinoma^9^,^20^, and renal cell carcinoma^21^.

PDTX models of gastric cancer using surgical tissues are reported occasionally^22^,^23^,^24^; however, patients with AGC are the most suitable population to evaluate the efficacy of new drugs, and the major method to acquire tumor samples for AGC is gastroscopic biopsy, especially for paired samples before and after chemotherapy. PDTX models using gastroscopic biopsies are best animal models to evaluate the efficacy of new drugs for AGC in preclinical studies, which will be presented in this study.

## Results

### Patient characteristics and establishment of PDTX model

A total of 185 patients were included in this study with 133 male (71.9%) and 52 female patients (28.1%) with a median age 60 years (25–80 years). The detailed characteristics of patients are shown in Table 1. Sixty-three PDTX models were successfully established (34.1%, 63/185), and the mean latency period of xenograft (from the day of inoculation to palpable tumor) was 65.86 ± 32.84 days (range: 11–160 days). No differences were observed between transplantation rate and characteristics except chemotherapy. Biopsies of prior chemotherapy had a higher transplantation rate (52.1%, 37/71) than biopsies after chemotherapy (22.8%, 26/114; *P* = 0.000; Table 1). In addition, no differences were found between the latency period of xenograft and characteristics of patients (Table 1 and Supplementary Fig. S1). Along with the increase in serial passage, the latency period was shorter and shorter (*P* = 0.000; Supplementary Fig. S1). Two patients in this study had paired samples before and after chemotherapy, and the matched PDTX models were also successfully established (case 039-1/-2 and case 093-1/-2).

### Histopathological characteristics of xenografts

Differentiation and Lauren classification of xenografts were judged and compared to primary tumors of patients by two independent pathologists. All PDTX models were compared and the histopathological features of PDTX were nearly consistent with those of primary tumors of patients. The concordance rate of differentiation between primary tumors of patients and xenografts was 90.5% (57/63), which was 98.4% (62/63) between different passages (P1, P2, and P3) of xenografts. Three patients with moderate differentiation of primary tumors changed to poor differentiation of xenografts (case 023, 027, and 144), one patient with poor differentiation of primary tumor changed to moderate differentiation of xenograft (case 009), one patient with moderate to poor differentiation of primary tumor changed to poor differenciation of xenograft (case 135), and one patient with poor differenciation of primary tumor changed to lymphoma of xenograft from P2 (case 070, Table 2).

**Figure 1:**
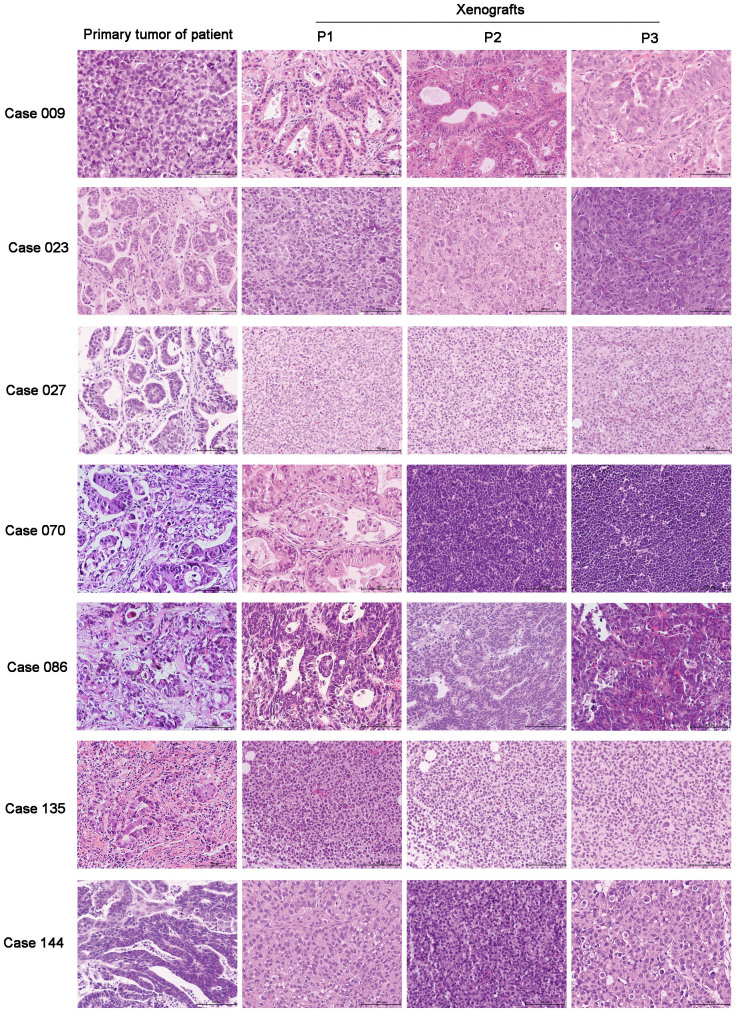
Disconcordance of Lauren classification between primary tumors and xenografts in 7 cases. Cases 023, 027, and 144 with intestinal type of primary tumors converted to diffuse type of xenografts; case 009 with diffuse type of primary tumor converted to intestinal type of xenograft; case 135 with mixed type of primary tumor converted to diffuse type of xenograft; case 086 with mixed type of primary tumor converted to intestinal type of xenograft; case 070 with mixed type of primary tumor converted to lymphoma of xenograft from P2. Scale bars, 100 μm.

The concordance rate of Lauren classification between primary tumors of patients and xenografts was 88.9% (56/63), which was 98.4% (62/63) between different passages (P1, P2, and P3) of xenografts. Three patients with intestinal type of primary tumors converted to diffuse type of xenografts (case 023, 027, and 144), one patient with diffuse type of primary tumor converted to intestinal type of xenograft (case 009), one patient with mixed type of primary tumor converted to diffuse type of xenograft (case 135), one patient with mixed type of primary tumor converted to intestinal type of xenograft (case 086), and one patient with mixed type of primary tumor converted to lymphoma of xenograft from P2 (case 070, Table 2 and Fig. 1).

For cases 039 (moderate-poor differenciation, intestinal type) and 093 (poor differentiation, diffuse type), the differentiation and Lauren classification were consistent between xenografts before and after chemotherapy (Supplementary Table S2 and Fig. S2a).

### Concordance of HER2 expression between primary tumors of patients and xenografts

**Figure 2:**
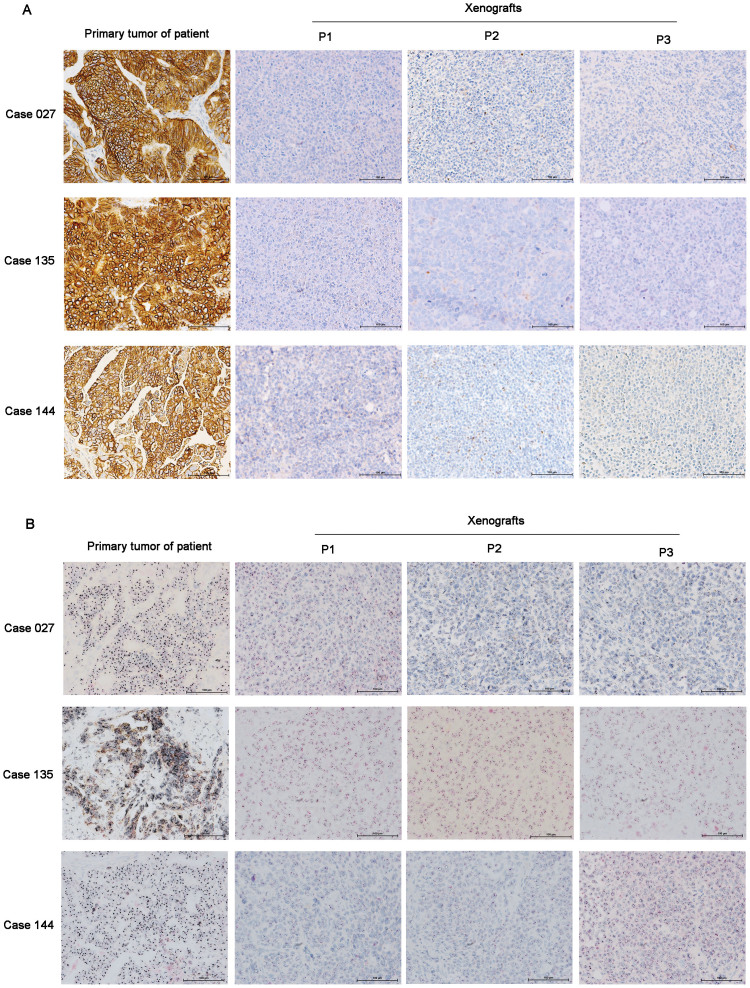
Disconcordance of HER2 expression between primary tumors and xenografts in 3 cases. Cases 027, 135, and 144 with HER2 positive expression of primary tumors converted to negative expression of xenografts based on IHC (a) and DISH (b) results. Scale bars, 100 μm.

As the only approved molecular target, HER2 expression was tested in all xenografts by IHC or DISH. Fifteen of 63 primary tumors of patients demonstrated HER2 positive expression (23.8%, IHC score 3+ or DISH amplification). The concordance rate of HER2 expression between primary tumors of patients and xenografts was 95.2% (60/63), which was 100% (63/63) between different passages (P1, P2, and P3) of xenografts. Cases 027, 135, and 144 with HER2 positive expression of primary tumors changed to negative expression of xenografts (Fig. 2) accompanied with changes of Lauren classification (cases 027 and 144 with intestinal type changed to diffuse type; case 135 with mixed type changed to diffuse type).

For cases 039 (HER2 negative) and 093 (HER2 positive), HER2 expression was consistent between xenografts before and after chemotherapy (Supplementary Fig. S2b).

### Chemosensitivity of PDTX models was comparable with patients

**Figure 3:**
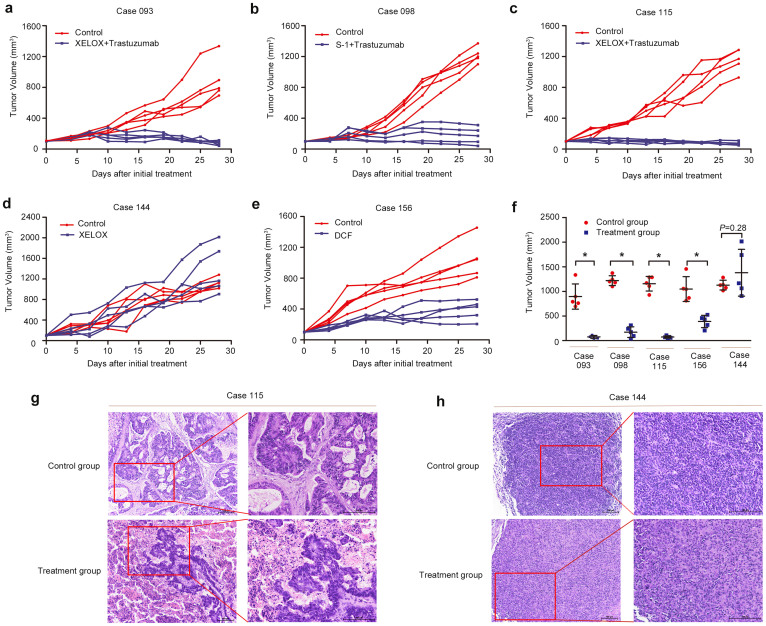
Therapeutic response of PDTX models treated with or without drugs. Four of the 5 PDTX models had comparable therapeutic responses with patients (a, b, c, and e), and case 144 had an inconsistent response with patient (d). After sacrificing the mice, significant differences of tumor volumes in cases 093, 098, 115, and 156 were found between control and treatment groups, which was not observed in case 144 (f). The percentage of tumor cells in xenografts that responded to chemotherapy was significantly decreased compared with control group (case 115, g), which was not seen in xenografts resistant to chemotherapy (case 144, h). Scale bars, 100 μm. Line and error bars represent mean and s.d. * *P*<0.01 according to unpaired two-tailed *t*-test.

One of the most important elements to evaluate the PDTX models is the therapeutic response. Five PDTX models were used in this study to compare the chemosensitivity in patients treated with first-line regimens of XELOX + Trastuzumab (n = 2), S-1 + Trastuzumab (n = 1), XELOX (n = 1), and DCF (n = 1; Table 3). When tumor volume of P4 animals reached approximately 150 mm^3^, 10 mice were randomized into two groups treated with above regimens or control for two cycles. The tumor size was measured and the therapeutic response was compared in the patients. Four of 5 PDTX models had comparable therapeutic responses (Table 3 and Fig. 3). One patient (case 144) demonstrated stable disease after treatment with XELOX; however, the PDTX model did not have any response to the XELOX regimen (Fig. 3). The disconcordance of therapeutic response between the PDTX model of case 144 and the patient might be due to changes in the Lauren classification. Case 144 with intestinal type of primary tumor converted to diffuse type of the PDTX model (Fig. 1). Further studies also indicated that compared to the control group, a large number of tissue necrosis was found in xenografts that responded to chemotherapy (case 115, Fig. 3g), which did not observed in xenografts resistance to chemotherapy (case 144, Fig. 3h). Moreover, despite a large number of tissue necrosis in xenografts after chemotherapy, a small proportion of residual tumor cells was viable and might be the basis of tumor recurrence and resistance.

### Genetic features were stable during serial passaging of PDTX models

To understand whether the genetic features of xenografts changed during the serial passaging of PDTX models, the entire exons of 265 cancer-associated genes were profiled by targeted next-generation sequencing. Fifteen samples from the 5 PDTX models (P2, P3, and P4 samples/PDTX model) were analyzed. Although the mutation profiles of some genes were variable during serial passaging of one PDTX model, the majority of genes were stable in all PDTX models (Supplementary Fig. S3).

## Discussion

PDTX models have become more popular in the last few years than the conventional models. PDTX models from various tumors have been established, including gastric cancer^22^,^23^,^25^,^26^, which was mostly derived from surgical tumor tissues. In China, the majority of gastric cancer patients are diagnosed as advanced gastric cancer (AGC) and are not candidates for surgery^27^. In AGC patients, gastroscopic biopsies were performed to acquire tumor samples. Whether PDTX models using gastroscopic biopsies could be successfully established and had features of primary tumors of patients has not been reported, and it was demonstrated for the first time in this study.

A total of 185 fresh gastroscopic biopsies of gastric cancer were subcutaneously transplanted into NOD/SCID mice and 63 PDTX models were successfully established (34.1%), with a mean latency period of xenograft at 65.86 ± 32.84 days (11–160 days). Previous studies of PDTX models using surgical tissues of gastric cancer were consistent with ours^22^. The established PDTX models in this study could be serially passaged to maintain tumors *in vivo*. Biopsies prior to chemotherapy had a higher transplantation rate (52.1%, 37/71) than biopsies after chemotherapy (21.9%, 25/114; *P* = 0.000). The possible reason was that biopsies treated by therapeutic drugs contained more necrotic or scar tissues resulting in a decreased transplantation rate, which could be partially explained by our results (Fig. 3g). No differences were found between the latency period of xenografts and characteristics of patients; however, the latency period was shorter with any additional serial passage (Supplementary Fig. S1), which was similar to other PDTX models^21^.

The PDTX model was established to serve all preclinical studies better; therefore, histopathological and molecular features between xenografts and primary tumors were tested. The majority of xenografts maintained the histopathological features (histomorphology, differenciation, Lauren classification, and so on) of primary tumors with the exception of a small part of xenografts. Six PDTX models developed changes in differentiation and 7 PDTX models developed changes in Lauren classification compared to primary tumors (Table 2), the possible reason was the high heterogeneity of gastric cancer. Our result also demonstrated that changes in differentiation and Lauren classification during passaging were closely related. A novel finding in our study was case 070 with primary gastric adenocarcinoma, which converted into lymphoma during passaging (Fig. 1). The definite mechanism was unknown, but the same phenomenon was once reported in PDTX model for renal cell carcinoma^21^.

As the only approved molecular target, HER2 expression was tested and the concordance rate between primary tumors and xenografts was very high except in 3 cases. Three patients with HER2 positive expression of primary tumors converted to negative expression of xenografts (Fig. 2) accompanied with changes in Lauren classification. This result was consistent with a previous report that patients with intestinal type had higher HER2 expression than diffuse type^28^. Besides HER2, other potential targets in gastric cancer, such as c-MET, PD-1/PDL-1, and IGFR, will be detected in the following study.

Except for the expression of some molecules, the mutation profiles of 265 cancer-related genes were tested during passaging to understand the genetic stability. Results based on next-generation sequencing suggested that the genetic characteristics were stable during passaging of xenografts (Supplementary Fig. S3). Some disconcordance, especially for case 156, was still observed due to the high heterogeneity of gastric cancer, which was similar to other reports^29^,^30^ and will be validated in future large sample studies. In this study, the primary tumors of patients and P1 xenografts were not analyzed by next-generation sequencing because the amounts of primary tumors and P1 xenografts were very little and barely enough to passaging. In the continuing study, we would detect some specified gene mutations using DNAs extracted from little FFPE (formalin-fixed paraffin-embedded) tumors.

Finally, the therapeutic response was tested between primary tumors and xenografts using the same regimens. The results demonstrated that 4 of 5 PDTX models had comparable therapeutic responses with patients (Table 3 and Fig. 3). Although the therapeutic response of case 144 was different between the primary tumor and xenograft, the change in Lauren classification during transplantation could explain the result. The primary tumor of case 144 was intestinal type, but the xenograft used in study converted to diffuse type. The therapeutic response of all PDTX models in this study was being continued and the results were expectant.

In summary, PDTX models using gastroscopic biopsies in gastric cancer were successfully established and identified for the first time. The histopathological and molecular features as well as therapeutic response were highly consistent with primary tumors of patients, which could provide a most realistic model for developing new drugs and exploring the mechanisms of drug resistance, and therefore provide evidence for individual therapy.

## Methods

### Patients and tumor samples

From November 2012 to July 2014, 185 patients who had endoscopic biopsies with histologically confirmed gastric cancer in the gastrointestinal department of the Peking University Cancer Hospital were included in this study. The clinical data of patients were collected from their medical records. All patients gave their written informed consent for their tumor samples to be used for research. This study was approved by the medical ethics committee of Peking University Cancer Hospital and carried out in accordance with the approved guidelines.

### Establishment of PDTX models

Four fragments of fresh gastroscopic biopsies were obtained from one patient with approximately 2 × 2 × 2 mm^3^/fragments. All fragments from one patient were subcutaneously inoculated into one flank of a 6-week-old NOD/SCID mouse (Beijing HFK Bio-Technology Co., LTD, Beijing, China). Tumor growth was measured twice weekly using a vernier caliper. The established PDTX model was called passage 1 (P1). When the tumor size of P1 reached approximately 750 mm^3^, the tumor was separated and sliced into small fragments (approximately 3 × 3 × 3 mm^3^/fragment) and re-inoculated into mice to obtain the subsequent passages called P2, P3, P4, and so on. All procedures were performed under sterile conditions at BeiGene (Beijing) SPF facility and carried out in accordance with the Guide for the Care and Use of Laboratory Animals of the National Institutes of Health. This experiment was approved by the ethics committee of animal experiments of BeiGene (Beijing) Co., Ltd.

### H&E staining and HER2 immunohistochemistry

H&E staining was performed using H&E staining kit (C0105, Beyotime, China) according to the manufacture's instructions. Immunohistochemistry (IHC) stainings of HER2 was performed using anti-HER2/neu antibody (4B5, Roche, Basel, Switzerland) according to a previous report^31^. H&E staining and IHC staining were reviewed and scored according to the criteria reported previously^32^ by two independent pathologists who were blinded to this study.

### Dual-color in situ hybridization (DISH)

As the only approved molecular target, HER2 amplification was tested in xenografts and primary tumors of patients using Ventana HER2 dual-color ISH assay (DISH, BenchMark XT). Amplification of HER2 was defined as a ratio HER2/CEP17 ≥ 2.2, and the result was read by two independent specialists who were blinded to this study.

### Targeted next-generation sequencing and data analysis

Genomic DNA was extracted from fresh tumor samples of xenografts using QIAamp DNA Mini Kit (Lot. 51304, QIAGEN) according to the manufacturer's instructions. Approximately 3 microgram of DNA was used in the following next-generation sequencing. A panel containing 265 cancer-associated genes (OncoCap_265 kit, MyGenostics, Baltimore, MD) is shown in Supplementary Table S1^33^,^34^. The entire exons of 265 genes were specifically enriched and sequenced on Illumina HiSeq 2000 sequencer according to reported procedures^35^,^36^. The mutation profile was drawn using R software after filtering data based on following references: the mean coverage ≥100, mutation ratio ≥10, absence of mutation in 1000 Genomes Project, and nonsynonymous mutations.

### Evaluation of chemosensitivity of PDTX models

In this study, PDTX models of passage 4 (P4) were used to evaluate the chemosensitivity. When the tumor volume reached approximately 150 mm^3^, 10 mice were randomized into the following two groups with similar tumor volumes: treatment group (n = 5) and control group (n = 5). The control group was treated with vehicle (physiological saline), and the treatment group was treated with a regimen consistent with patient. Treatment regimens in this study included the following: XELOX (Capecitabine plus Oxaliplatin), XELOX + Trastuzumab, S-1 + Trastuzumab, and DCF (Docetaxel plus Cisplatin plus 5-Fluorouracil), the dosage of which was according to previous reports^37^,^38^,^39^. Mice were treated for 7 consecutive days with a 7-day interval of each cycle (two weeks per cycle) for 2 cycles. Tumor size and body weight were measured twice weekly, and the tumor volume (V) was calculated using the following formula: V = L × W^2^/2 (L, length, long diameter of tumor; W, width, short diameter of tumor). All animal procedures were approved by the ethics committee of animal experiments of the Peking University Cancer Hospital.

### Statistical analysis

Statistical analysis was performed using SPSS 17.0 software. The relationships between clinicopathological characteristics and transplantation rate or latency period of xenografts were analyzed using the chi-square test, unpaired two-tailed *t*-test or one-way analysis of variance (ANOVA). Tumor growth between two groups was compared using repeated-measured analysis of variance. *P* < 0.05 was considered statistically significant.

## Author Contributions

J.G. and L.S. conceived and designed the experiments; Y.Z., T.T., Z.T., L.W., Y.L., Y.L., N.L. and J.Z. performed the animal model construction and experiments; Z.L., J.W. and B.D. analyzed the data and contributed to writing and editing the manuscript; Y.Z. and J.G. wrote the manuscript. All authors discussed the results and commented on the manuscript.

## Supplementary Material

Supplementary Information Supplementary Information

## Figures and Tables

**Table 1:** Patient characteristics, transplantation rate, and latency period of xenografts

Characteristics	No. of patients (%)	Latency period (days)	*P*	Transplantation rate (%)	*P*
Gender			0.5		0.92
Male	133 (71.2%)	67.05 ± 34.40		33.8% (45/133)	
Female	52 (28.1%)	63.00 ± 29.52		34.6% (18/52)	
Age (years)			0.672		0.724
≤45	25 (13.5%)	73.88 ± 32.16		32.0% (8/25)	
45–60	69 (37.3%)	67.17 ± 31.55		37.7% (26/69)	
≥60	91 (49.2%)	62.37 ± 34.78		31.9% (29/91)	
Stage			0.682		0.827
I/II	5 (27.0%)	47.5 ± 14.85		40.0% (2/5)	
III/IV	173 (93.5%)	67.16 ± 33.13		35.3% (61/173)	
NA	7 (37.8%)	57		14.3% (1/7)	
Primary site			0.893		0.653
Upper	52 (28.1%)	63.18 ± 29.90		38.5 (20/52)	
Middle	62 (33.5%)	70.24 ± 35.86		35.5 (22/62)	
Lower	65 (33.1%)	63.16 ± 33.96		30.8 (20/65)	
Anastomosis	6 (32.4%)	71		16.7 (1/6)	
Differentiation			0.18		0.212
High	3 (1.6%)	13		33.3 (1/3)	
Moderate	59 (31.9%)	59.36 ± 31.15		42.4 (25/59)	
Moderate-poor	42 (22.7%)	67.27 ± 29.82		38.1 (16/42)	
Poor	81 (43.8%)	74.60 ± 35.07		25.9 (21/81)	
Lauren classification			0.277		0.291
Intestinal	89 (48.1%)	59.58 ± 30.76		39.3% (35/89)	
Diffuse	79 (42.7%)	74.43 ± 34.19		27.8% (22/79)	
Mixed	17 (9.2%)	68.33 ± 37.16		35.3% (6/17)	
HER2 expression			0.385		0.603
Negative	145 (78.4%)	61.27 ± 28.15		33.1% (48/145)	
Positive	40 (21.6%)	68.20 ± 34.43		37.5% (15/40)	
Chemotherapy			0.505		0.000
Before	71 (38.4%)	67.85 ± 34.33		52.1% (37/71)	
After	114 (61.6%)	63.30 ± 31.77	0.782	22.8% (26/114)	0.6
Partial response	21 (18.4%)	73.14 ± 35.81		33.3% (7/21)	
Stable disease	39 (34.2%)	62.67 ± 20.91		17.9% (7/39)	
Progressive Disease	49 (43.0%)	56.80 ± 35.32		22.4% (11/49)	
NA	5 (4.4%)	57		20.0% (1/5)	

Note: NA, non-available. Data represent mean ± s.d. *P* calculated by chi-square test, unpaired two-tailed *t*-test or one-way analysis of variance separately.

**Table 2:** Disconcordance of differentiation and Lauren classification of primary tumors of patients and xenografts

Case	Patient		Xenograft					
	Primary tumor		P1		P2		P3	
	Differenciation	Lauren	Differenciation	Lauren	Differenciation	Lauren	Differenciation	Lauren
023	Moderate	Intestinal	Poor	Diffuse	Poor	Diffuse	Poor	Diffuse
027	Moderate	Intestinal	Poor	Diffuse	Poor	Diffuse	Poor	Diffuse
144	Moderate	Intestinal	Poor	Diffuse	Poor	Diffuse	Poor	Diffuse
009	Poor	Diffuse	Moderate	Intestinal	Moderate	Intestinal	Moderate	Intestinal
135	Moderate-poor	Mixed	Poor	Diffuse	Poor	Diffuse	Poor	Diffuse
086	Moderate-poor	Mixed	Moderate-poor	Intestinal	Moderate-poor	Intestinal	Moderate-poor	Intestinal
070	Moderate-poor	Mixed	Moderate-poor	Mixed	Lymphoma	Lymphoma	Lymphoma	Lymphoma

**Table 3:** Therapeutic response of patients and PDTX models

Case	Stage	Differentiation	Lauren	HER2 expression	Regimen	Clinical response of patients	Concordance^a^
093	IV	Poor	Diffuse	Positive	XELOX+Trastuzumab	PR	Yes
098	IV	Well	Intestinal	Positive	S-1+Trastuzumab	SD	Yes
115	IV	Moderate	Intestinal	Positive	XELOX+Trastuzumab	PR	Yes
144	IV	Moderate	Intestinal	Positive	XELOX	SD	No
156	IV	Moderate	Intestinal	Negative	DCF	Increased SD	Yes

Note: PR, partial response; SD, stable disease;

^a^concordance: concordance of therapeutic response between patients and PDTX models.
