# Supplementary material for: Establishment and characterization of patient-derived tumor xenograft using gastroscopic biopsies in gastric cancer
Source: Sci Rep. 2015 Feb 25;5:8542. doi: 10.1038/srep08542 (PMC4339807; doi:10.1038/srep08542)
Supplement: Supplementary Information [file srep08542-s1.doc]

**Establishment and characterization of patient-derived tumor xenograft using** **gastroscopic biopsies in gastric cancer**

Yan Zhu^*,1^, Tiantian Tian^*,1^, Zhongwu Li^2^, Zhiyu Tang^3^, Lai Wang^3^, Jian Wu^4^, Yilin Li^1^, Bin Dong^2^, Yanyan Li^1^, Na Li^1^, Jianling Zou^1^, Jing Gao^#,1^, Lin Shen^#,1^

^1^Department of Gastrointestinal Oncology, ^2^Department of pathology, Key laboratory of Carcinogenesis and Translational Research (Ministry of Education), Peking University Cancer Hospital and Institute, Beijing, China

^3^BeiGene (Beijing) Co., Ltd, China

^4^MyGenostics Inc. Beijing, China

*****These authors contributed equally to this study.

**Correspondence to:**

Professor Lin Shen, Fu-Cheng Road 52, Hai-Dian District, Beijing 100142, China. Tel: +86-10-88196561; Fax: +86-10-88196561; Email: [lin100@medmail.com.cn](mailto:lin100@medmail.com.cn); or Professor Jing Gao, Fu-Cheng Road 52, Hai-Dian District, Beijing 100142, China. Tel: +86-10-88196747; Email:

[gaojing_pumc@163.com](mailto:gaojing_pumc@163.com)

**
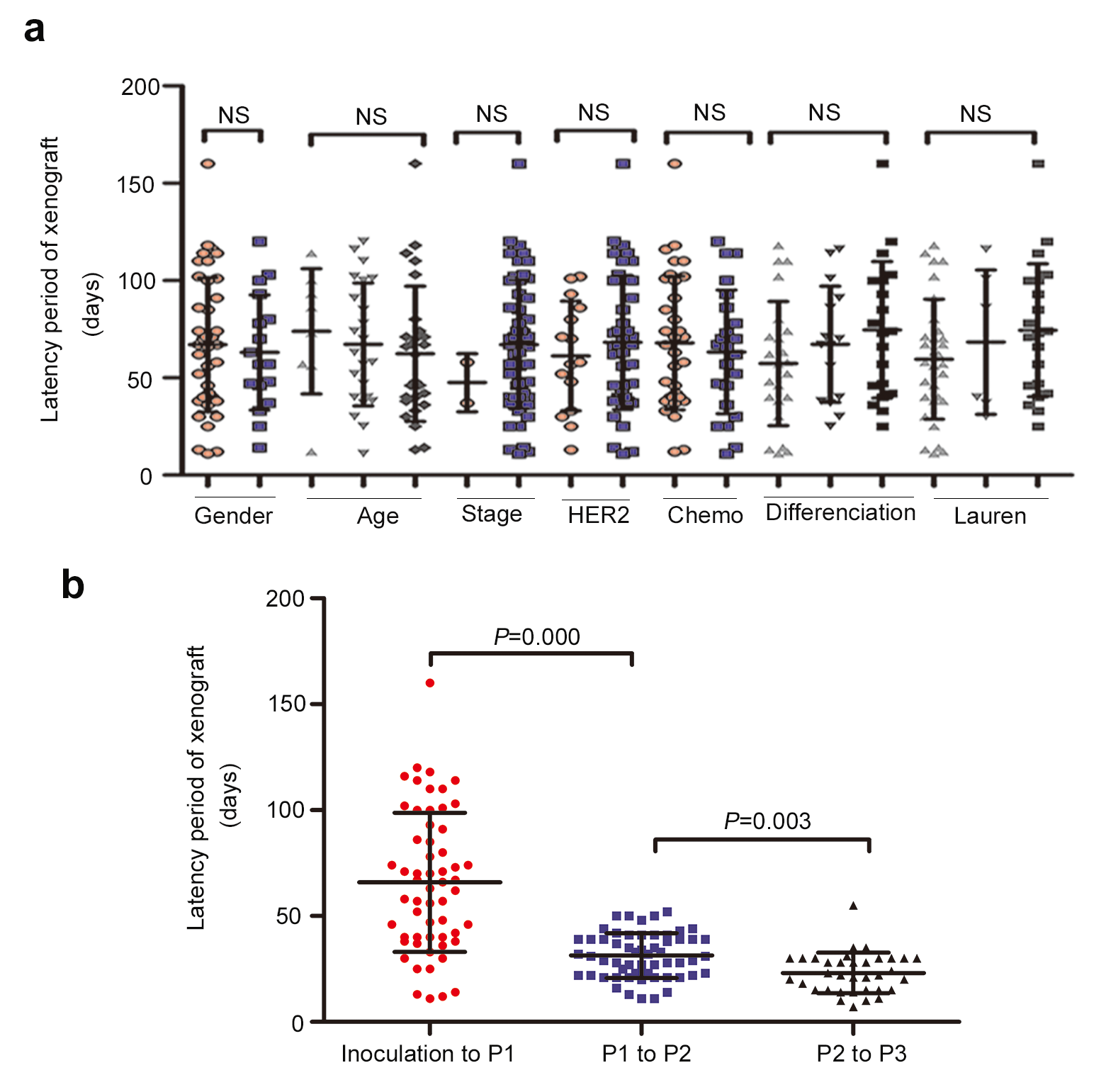
**

**Supplementary Figure S1.** **The latency period of xenografts.** (a) No significant differences were found between latency period of xenografts and characteristics of patients (from left to right: male, female, ≤45 years, 45-60 years, ≥60 years, stage I/II, stage III/IV, HER2 positive, HER2 negative, before chemotherapy, after chemotherapy, high-moderate differenciation, moderate-poor differenciation, poor differenciation, intestinal type, mixed type, diffuse type). (b) With increases in serial passage, the latency period became shorter and shorter (*P*=0.000). Line and error bars represent mean and s.d. NS, no significant; *P* calculated by unpaired two-tailed *t*-test or one-way analysis of variance.

**
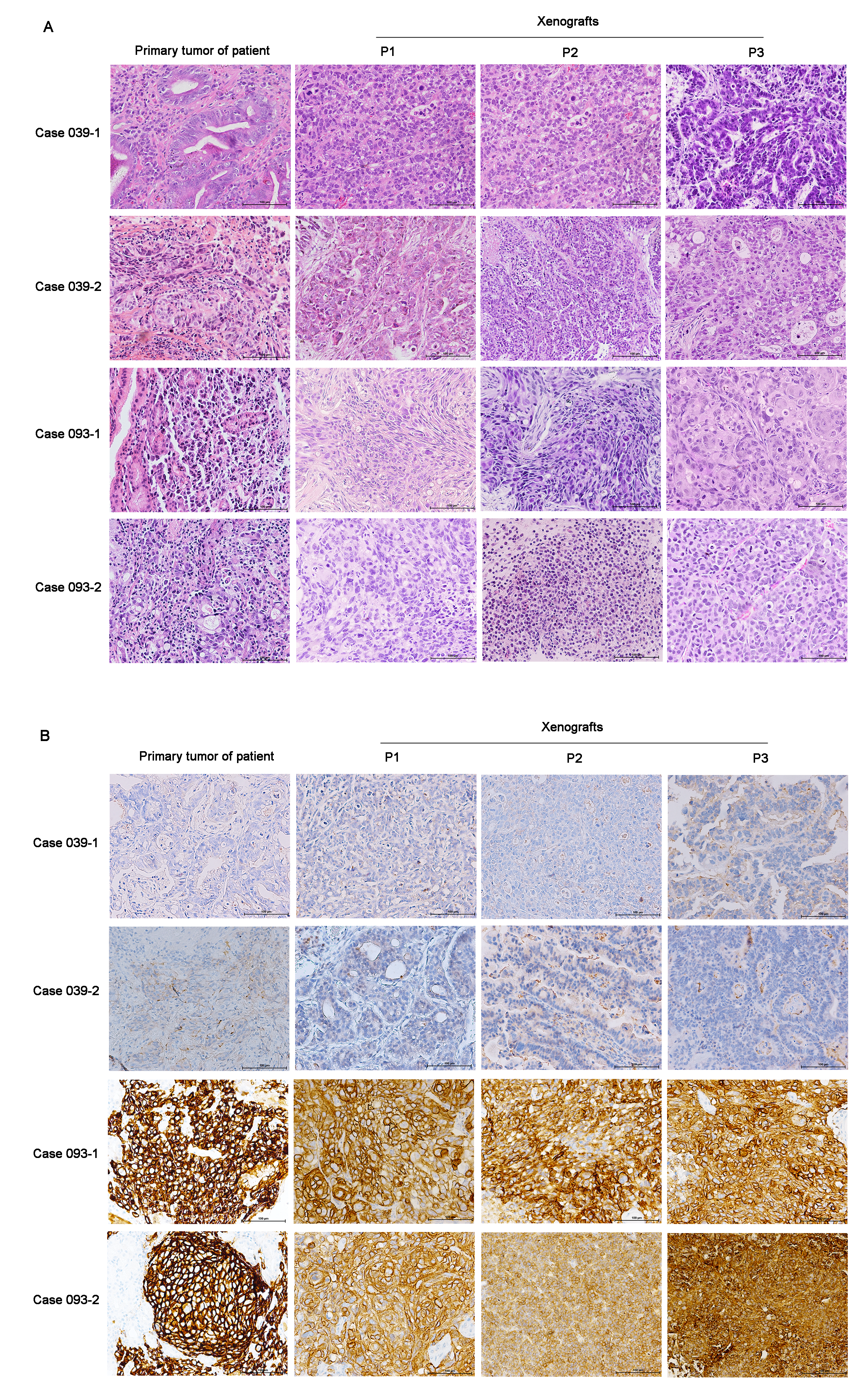
**

**Supplementary Figure S2.** **Histopathological characteristics and HER2 expression in cases with paired samples before and after chemotherapy.** The differentiation and Lauren classification (a) and HER2 expression (b) were consistent between xenografts of before and after chemotherapy. (case 039-1 and case 093-1: before chemotherapy; case 039-2 and case 093-2: after chemotherapy).

**
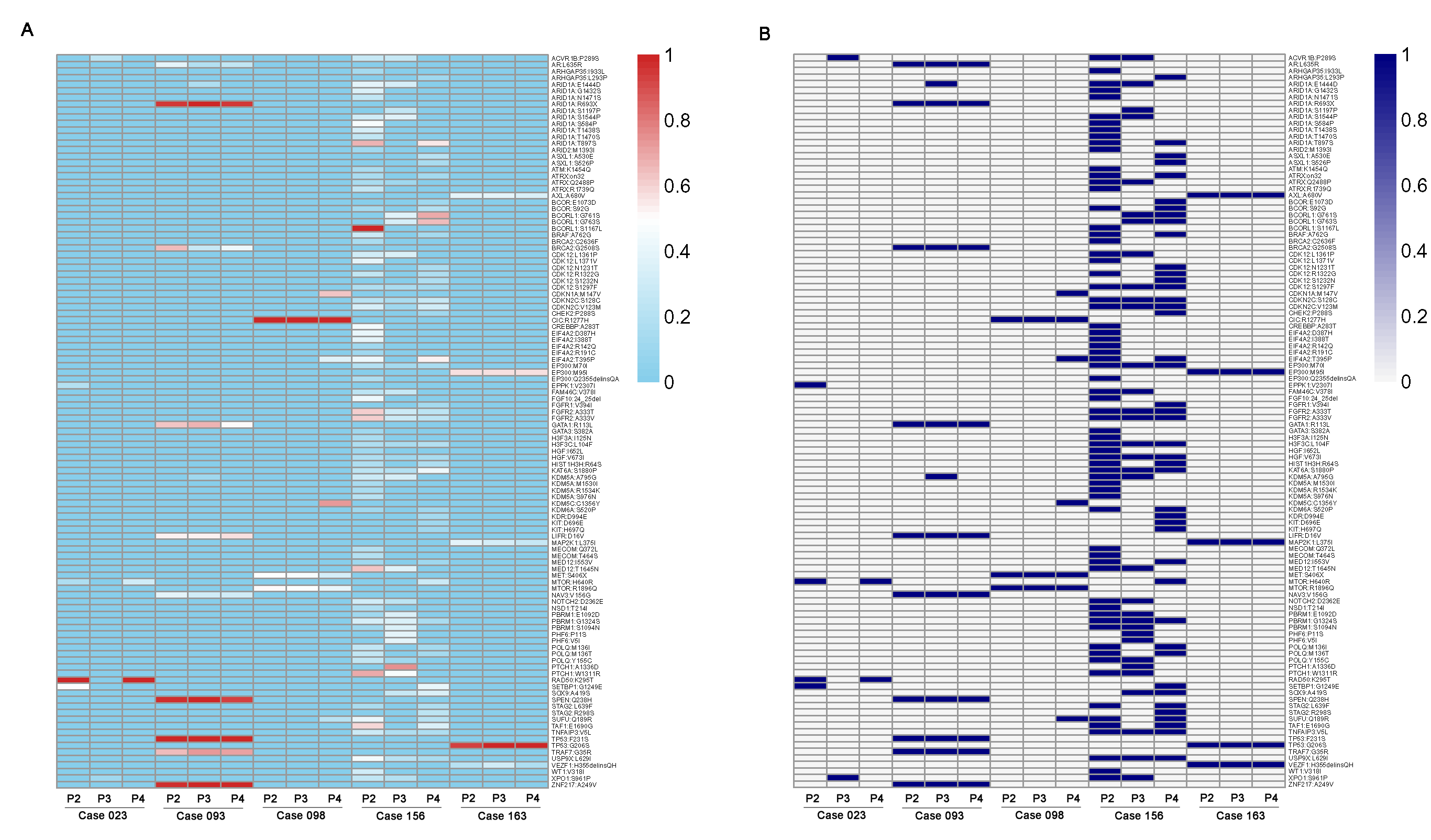
**

**Supplementary Figure S3.** **Mutation profile of 265 cancer-related genes during passaging.** (a) The profiling of mutant genes among different passages if mutation ratio was considered. (b) The profiling of mutant genes among different passages if only presence or absence of mutation was considered.

**Supplementary Table S1. The list of 265 cancer-associated genes.**

| ABL1 | EGR3 | MED12 | SMARCB1 | FANCL |
| --- | --- | --- | --- | --- |
| ACVR1B | EIF4A2 | MEN1 | SMC1A | FGF10 |
| ACVR2A | ELF3 | MET | SMC3 | FGF14 |
| AJUBA | EP300 | MIR142 | SMO | FGF19 |
| AKT1 | EPHA3 | MLH1 | SOCS1 | PDK1 |
| ALK | EPHB6 | MLL2 | SOX17 | PIK3R2 |
| APC | EPPK1 | MLL3 | SOX9 | RAD50 |
| AR | ERBB2 | MLL4 | SPOP | RAD51 |
| ARHGAP35 | ERBB4 | MPL | SRSF2 | BTK |
| ARID1A | ERCC2 | MSH2 | STAG2 | CD79A |
| ARID1B | EZH2 | MSH6 | STK11 | CD79B |
| ARID2 | FAM123B | MTOR | TAF1 | CDKN2B |
| ARID5B | FBXW7 | MYD88 | TBL1XR1 | MUTYH |
| ASXL1 | FGFR2 | NAV3 | TBX3 | NFKBIA |
| ATM | FGFR3 | NCOR1 | TET2 | NTRK2 |
| ATR | FLT3 | NF1 | TGFBR2 | NUP93 |
| ATRX | FOXA1 | NF2 | TLR4 | PALB2 |
| AXIN1 | FOXA2 | NFE2L2 | TNFAIP3 | FGF23 |
| AXIN2 | FOXL2 | NFE2L3 | TP53 | FGF3 |
| B2M | FUBP1 | NOTCH1 | TRAF7 | FGF4 |
| B4GALT3 | GATA1 | NOTCH2 | TSC1 | FGF6 |
| BAP1 | GATA2 | NPM1 | TSHR | GID4 |
| BCL2 | GATA3 | NRAS | TSHZ2 | GNA13 |
| BCOR | GNA11 | NSD1 | TSHZ3 | GSK3B |
| BRAF | GNAQ | PAX5 | U2AF1 | SPEN |
| BRCA1 | GNAS | PBRM1 | USP9X | STAT4 |
| BRCA2 | H3F3A | PCBP1 | VEZF1 | SUFU |
| CARD11 | H3F3C | PDGFRA | VHL | TNFRSF14 |
| CASP8 | HGF | PHF6 | WT1 | WISP3 |
| CBFB | HIST1H1C | PIK3CA | ARAF | XPO1 |
| CBL | HIST1H2BD | PIK3CG | AXL | ZNF217 |
| CCND1 | HIST1H3B | PIK3R1 | BARD1 | ZNF703 |
| CDC73 | HNF1A | POLQ | BCORL1 | CCND1 |
| CDH1 | HRAS | PPP2R1A | BLM | CDKN2C |
| CDK12 | IDH1 | PRDM1 | BRIP1 | IKZF1 |
| CDKN1A | IDH2 | PRX | IL7R | LMO1 |
| CDKN1B | JAK1 | PTCH1 | IRF4 | MAP2K4 |
| CDKN2A | JAK2 | PTEN | JUN | MDM2 |
| CDKN2C | JAK3 | PTPN11 | KAT6A | MDM4 |
| CEBPA | KDM5C | RAD21 | KDM5A | MYC |
| CHEK2 | KDM6A | RB1 | KLHL6 | MYCL1 |
| CIC | KEAP1 | RET | MEF2B | MYCN |
| CREBBP | KIT | RNF43 | DDR2 | NCOA3 |
| CRIPAK | KLF4 | RPL22 | DOT1L | NKX2-1 |
| CRLF2 | KRAS | RPL5 | EMSY | SKP2 |
| CSF1R | LIFR | RUNX1 | ERG | FGFR1 |
| CTCF | LRRK2 | SETBP1 | FAM46C | KDR |
| CTNNB1 | MALAT1 | SETD2 | FANCA | TSC2 |
| CYLD | MAP2K1 | SF3B1 | FANCC | CDK6 |
| DAXX | MAP2K4 | SIN3A | FANCD2 | CDK4 |
| DNMT1 | MAP3K1 | SMAD2 | FANCE | CCNE1 |
| DNMT3A | MAPK8IP1 | SMAD4 | FANCF | E2F3 |
| EGFR | MECOM | SMARCA4 | FANCG | AURKA |

**Supplementary Table S2. Differentiation and Lauren classification of patients with paired samples before and after chemotherapy.**

| Case | Patient | | Xenograft | | | | | |
| --- | --- | --- | --- | --- | --- | --- | --- | --- |
|  | Primary tumor | | P1 | | P2 | | P3 | |
|  | Differenciation | Lauren | Differenciation | Lauren | Differenciation | Lauren | Differenciation | Lauren |
| 039-1 | Moderate-poor | Intestinal | Moderate-poor | Intestinal | Moderate-poor | Intestinal | Moderate-poor | Intestinal |
| 039-2 | Moderate-poor | Intestinal | Moderate-poor | Intestinal | Moderate-poor | Intestinal | Moderate-poor | Intestinal |
| 093 | Poor | Diffuse | Poor | Diffuse | Poor | Diffuse | Poor | Diffuse |
| 093-2 | Poor | Diffuse | Poor | Diffuse | Poor | Diffuse | Poor | Diffuse |
